# Evaluating the Association of Ki-67 with Oncotype DX Recurrence Score in Early-Stage ER-Positive/HER2-Negative Breast Cancer

**DOI:** 10.3390/cancers18111731

**Published:** 2026-05-26

**Authors:** Dimitrios Dragoumis, George Kapetsis, Konstantinos Louis, Dimitrios Maniatis, Eleni Mpalampou, Konstantinos Bouloukos, Xenophon Xenakis, Nikolaos Papaioannou, Styliani Parpoudi, Grigorios Pesmatzoglou, Anna Sachoulidou, Eleftherios Sfakianakis, Sofia Triantafyllidou, Vlasios Tsantilas, Aris Tsiftsoglou, Sofia Filippidou, Ioannis P. Fyssas, Maroulio Stathoulopoulou, Maria Matiatou, Panagiotis Karathanasis, Dimitrios Alexandrou, Anastasia Amanatidou, Klearchos Desiris, Eleni Efraimidou, Apostolos Zavos, Evropi Michailidou, Sotirios Roussogiannis, Vasileios Venizelos

**Affiliations:** 1 Breast Division, Department of General Surgery, St. Luke’s Hospital, Panorama, 55236 Thessaloniki, Greece; ddragoumis@gmail.com (D.D.); anastasia.aman@gmail.com (A.A.); 2 Genekor Medical S.A., 15344 Athens, Greece; 3 Third Department of Obstetrics and Gynecology, University General Hospital “ATTIKON”, Medical School, National and Kapodistrian University of Athens, 12462 Athens, Greece; kostaslouisss@gmail.com; 4 Second Breast Clinic, General Maternity and Gynecology Clinic, IASO General Hospital, 15123 Athens, Greece; maniatisdimitris.md@gmail.com (D.M.); sfilippidou18@gmail.com (S.F.); 5 Second Department of Surgical Oncology, General Anticancer and Oncology Hospital of Athens “Agios Savvas”, Alexandras Avenue 171, 11522 Athens, Greece; mpalampoueleni@gmail.com; 6 First Breast Clinic, General Maternity and Gynecology Clinic, IASO General Hospital, 15123 Athens, Greece; kbouloukos@gmail.com; 7 Second Surgical Breast Department, Henry Dunant Hospital Center, 11526 Athens, Greece; drxenakisx@gmail.com; 8 Surgical Department, Patsidis Clinic, 41335 Larissa, Greece; papnik-doc@hotmail.com; 9 Surgical Breast Oncology Department, Theageneio Hospital, 54639 Thessaloniki, Greece; stellaparpoudi@gmail.com; 10 Surgical Breast Clinic, Εugenideio Hospital, 11528 Athens, Greece; iatreiomastou@gmail.com; 11 Second Department of Propaedeutic Surgery, School of Medicine, Faculty of Health Sciences, Hippokration General Hospital of Thessaloniki, Aristotle University of Thessaloniki, 54124 Thessaloniki, Greece; a.sachoulidou@gmail.com; 12 Breast Clinic, 71202 Heraklion, Greece; elsfakianakis@windowslive.com; 13 Breast Clinic, Genesis Hospital, 54301 Thessaloniki, Greece; sofiatrianta@gmail.com; 14 Department of Breast and Plastic Surgery, 424 General Army Hospital, 56429 Thessaloniki, Greece; tsantilas_vl@hotmail.com; 15 Department of Surgery, St. Luke’s Hospital, Panorama, 55236 Thessaloniki, Greece; aristsiftsoglou@gmail.com (A.T.); kdesiris@doctors.org.uk (K.D.); 16 Prolipsis Medical Diagnostic Centre, 11528 Athens, Greece; john@fyssas.gr; 17 ΕUSOMA-Certified Multidisciplinary Breast Center, Metropolitan Hospital, 18547 Piraeus, Greece; maroulio.stath@gmail.com (M.S.); m.matiatou@gmail.com (M.M.); pkarathanasis@yahoo.gr (P.K.); bennievenizelos@gmail.com (V.V.); 18 First Department of Surgery, General Hospital Papageorgiou, Aristotle University of Thessaloniki, 56429 Thessaloniki, Greece; dimalexthess@gmail.com; 19 First Department of Surgery, University Hospital of Alexandroupolis, 68100 Alexandroupolis, Greece; eeffraem@med.duth.gr; 20 University Gynecological Clinic, University of Thessaly, 41500 Larissa, Greece; zavosa@gmail.com; 21 Department of General Surgery, Agios Pavlos General Hospital, 55134 Thessaloniki, Greece; europi2@yahoo.gr; 22 Surgical Clinic, Elpis-Papaioannou General Clinic, 38221 Volos, Greece; roussotos@gmail.com

**Keywords:** Ki-67, oncotype DX, recurrence score, hormone receptor-positive breast cancer, HER2-negative breast cancer, tumor proliferation, genomic assays, adjuvant therapy decision-making, early-stage breast cancer

## Abstract

**Background/Objectives**: Ki-67 is widely used as an immunohistochemical marker of tumor proliferation in hormone receptor-positive (HR-positive), HER2-negative breast cancer, but its interpretation is limited by variability and uncertain concordance with genomic assays. The Oncotype DX^®^ Recurrence Score (RS) is a validated multigene assay with established prognostic and predictive utility. This study evaluated the relationship between Ki-67 and RS in clinical practice. **Methods**: We retrospectively analyzed women in Greece with early-stage estrogen receptor-positive, HER2-negative breast cancer without distant metastasis (pM0) who underwent Oncotype DX testing between 2020 and 2023. Eligible patients were node-negative or postmenopausal with node-positive disease. RS was categorized as low (0–25) or high (>25). Ki-67 was assessed using binary (<20% vs. ≥20%) and three-tier (≤5%, >5–<30%, ≥30%) classifications. Associations were analyzed using correlation, concordance, and non-parametric methods. **Results**: Among 2967 patients, the median RS was 16, with similar distributions across nodal subgroups. Ki-67 and RS demonstrated a modest positive correlation as continuous variables (R = 0.30, *p* < 0.001). After stratification, associations with RS were observed only in tumors with high Ki-67 expression, whereas no correlation was detected in low or intermediate groups. RS distributions differed significantly across Ki-67 strata. Overall concordance between Ki-67-based proliferation categories and RS-based genomic risk was 56.2%, with discordant cases in both directions. **Conclusions**: Ki-67 shows a modest association with Oncotype DX RS, but substantial discordance indicates Ki-67 should not substitute genomic testing in HR-positive/HER2-negative early breast cancer.

## Simple Summary

Doctors caring for patients with early-stage breast cancer often rely on tests that estimate how actively cancer cells are growing to help decide whether chemotherapy is needed. One commonly used test examines tumor samples under a microscope, while another analyzes the activity of many genes within the cancer. These tests are sometimes thought to provide similar information, but how well they agree in everyday clinical practice is unclear. In this study, we examined data from a large group of women in Greece with hormone receptor-positive, HER2-negative early breast cancer who underwent gene-based tumor testing. We found that the simple laboratory marker and the gene-based test often gave different results for individual patients. This highlights the importance of genomic testing for personalized treatment decisions and supports current medical guidance against using simpler tests as substitutes.

## 1. Introduction

Breast cancer is the most frequently diagnosed malignancy worldwide and a leading cause of cancer-related mortality [1]. Owing to its biological heterogeneity, accurate tumor characterization is essential for prognostic assessment and therapeutic decision making.

In routine clinical practice, this characterization relies on a standardized panel of biomarkers, including mainly estrogen receptor (ER), progesterone receptor (PR), and human epidermal growth factor receptor 2 (HER2), along with the Ki-67 proliferation index, which together inform tumor subtype assignment, prognosis, and selection of systemic therapies [2,3,4].

Among these biomarkers, Ki-67 is a nuclear protein expressed during active phases of the cell cycle and serves as a marker of cellular proliferation. In hormone receptor-positive, HER2-negative breast cancer, higher Ki-67 expression has been consistently associated with increased tumor aggressiveness, higher histologic grade, and worse clinical outcomes [5,6,7].

Ki-67 immunohistochemical expression has long been used as a surrogate marker of tumor proliferative activity in breast cancer; however, the optimal cut-off values for clinical interpretation have evolved over time. Early prognostic studies in the late 1990s and early 2000s commonly applied low Ki-67 thresholds around 10%, showing that increased proliferative activity was associated with inferior disease-free and overall survival, albeit with substantial inter-study variability due to differences in assessment methodology and scoring [8].

The 2011 St Gallen Consensus proposed a Ki-67 cut-off of 14% to distinguish luminal A-like from luminal B-like tumors when immunohistochemistry was used as a surrogate for intrinsic molecular subtyping, based on correlations with gene expression profiling [9]. However, concerns regarding limited reproducibility and inter-laboratory variability prompted reassessment in the 2013 St Gallen Consensus, which suggested that a higher threshold of approximately 20% might better identify tumors with increased proliferative activity [10]. Subsequent studies supported this shift, demonstrating improved concordance between Ki-67 ≥ 20% and genomic risk signatures, leading to its broad adoption as a marker of high proliferation in hormone receptor-positive breast cancer and its incorporation into clinical practice and trial designs [11,12].

The clinical relevance of a Ki-67 cut-off ≥20% was further highlighted by the monarchE trial, in which this threshold, together with other clinicopathologic criteria, was used to classify patients with 1–3 positive axillary lymph nodes (N1) as having high risk disease [13]; although subsequent analyses demonstrated Ki-67 to be prognostic but not predictive of abemaciclib benefit, leading to removal of this criterion from mandatory eligibility requirements [14,15].

More recently, recognition of Ki-67 as a continuous biological variable, rather than a dichotomous marker, has shaped contemporary guideline recommendations. The International Ki-67 in Breast Cancer Working Group (IKWG) proposed a three-tier framework to improve interpretability while accounting for analytic variability: very low values (≤5%), very high values (≥30%), and an intermediate range (5–30%) [16]. Within this framework, Ki-67 provides meaningful prognostic information at the extremes, whereas intermediate values should not guide treatment decisions in isolation and require integration with clinicopathologic factors and/or genomic assays.

Despite its widespread use, the clinical interpretation of Ki-67 remains challenging due to substantial inter-observer and inter-laboratory variability, differences in pre-analytical and analytical handling, heterogeneity in scoring methodologies, and the lack of universally accepted cut-off values [16,17,18,19,20,21,22]. These limitations are consistently acknowledged across all major international guidelines and consensus statements, including St Gallen, ESMO, ASCO, and the International Ki-67 in Breast Cancer Working Group [11,16,23,24].

Although Ki-67 is a well established prognostic marker in ER-positive/HER2-negative breast cancer, evidence supporting its predictive ability for chemotherapy benefit remains inconsistent and insufficient for clinical decision making.

In a systematic review and meta-analysis of 53 studies including 10,848 patients, pre-treatment Ki-67 was associated with higher pathological response rates to neoadjuvant chemotherapy in breast cancer, regardless of HR-positive, HER2-positive, or triple-negative disease subtype [25]. Similar trends have been reported in additional studies across all breast cancer molecular subtypes; however, the use of heterogeneous Ki-67 cut-off values across these studies has raised further questions regarding the robustness and clinical applicability of Ki-67 [26,27,28,29].

However, several studies have failed to demonstrate a correlation between Ki-67 and pathological complete response [30,31], and while pCR is strongly associated with favorable long-term outcomes in HER2 positive and triple negative breast cancer, this relationship is less evident in HR+/HER2− breast cancer, where pCR is less common.

In prospective–retrospective analyses of the randomized IBCSG VIII and IX trials, in which patients were assigned to chemotherapy plus endocrine therapy versus endocrine therapy alone, a high Ki-67 labeling index was associated with worse disease-free survival across all treatment arms. Importantly, no interaction between Ki-67 expression and treatment assignment was observed, indicating that the effect of treatment on disease-free survival was independent of Ki-67 status and supporting a prognostic rather than predictive role for Ki-67 [32].

Given the insufficient evidence to support Ki-67 as a predictive marker for adjuvant chemotherapy benefit, International Ki-67 in Breast Cancer Working Group and ASCO have concluded that Ki-67 has established clinical validity as a prognostic biomarker but should not be used alone to guide chemotherapy decisions in ER-positive/HER2-negative breast cancer [16,23].

These limitations have prompted increasing interest in multigene expression assays that offer standardized and reproducible assessments of tumor biology.

Oncotype DX is a validated multigene expression assay that characterizes tumor biology by analyzing the expression of 21 genes related to proliferation, hormone receptor signaling, HER2 signaling, invasion, and other biologic processes, generating a Breast Recurrence Score^®^ (RS) ranging from 0 to 100. Ki-67 (MKI-67) constitutes a key component of the proliferation gene module, which contributes substantially—but not exclusively—to the overall Recurrence Score. In patients with early-stage, hormone receptor-positive, HER2-negative breast cancer, the assay has been extensively validated in both retrospective and prospective studies as a prognostic marker of recurrence risk and a predictive tool for chemotherapy benefit, including in patients with node-negative disease and those with up to three positive axillary lymph nodes [33,34,35,36,37,38,39,40]. On the basis of results from pivotal prospective trials, TAILORx and RxPONDER, the Oncotype DX Recurrence Score has been integrated into international treatment algorithms to guide adjuvant chemotherapy decision making [33,35]. According to NCCN guidelines, in postmenopausal patients or those aged ≥50 years with node-negative (N0) or limited node-positive (N1, 1–3 positive nodes) disease, a Recurrence Score (RS) of 0–25 supports omission of adjuvant chemotherapy, whereas scores > 25 identify patients for whom chemotherapy is recommended. In premenopausal patients with node-negative disease, no benefit has been observed from the addition of chemotherapy to standard endocrine therapy for RS 0–15, while RS 16–25 has been associated with a trend towards absolute benefit from chemotherapy in addition to standard endocrine therapy, as demonstrated in pre-specified subgroup analyses of the TAILORx trial, and RS ≥ 26 indicate a clear recommendation for adjuvant chemotherapy [41].

Given that Ki-67 and the Recurrence Score both capture aspects of tumor proliferative activity, evaluating their relationship may provide insight into the concordance and potential added value of these biomarkers.

In a systematic review and meta-analysis encompassing 18 studies with sample sizes ranging from 53 to 4695 (median sample size, 106), Ki-67 demonstrated a positive association with the Oncotype DX Recurrence Score in hormone receptor-positive, HER2-negative early breast cancer, with the exception of two studies [42]. However, pronounced inter-study heterogeneity, together with substantial methodological variability, including the use of heterogeneous cut-off values for both Ki-67 expression and Recurrence Score risk categories—limited its ability to reliably predict Recurrence Score categories, reinforcing that Ki-67 cannot act as substitute for multigene assays. Consistent with this meta-analysis, additional studies in the literature have yielded conflicting results regarding the relationship between Ki-67 and the Oncotype DX Recurrence Score, with some reporting a positive association [43,44,45,46,47] while others failed to demonstrate a significant correlation [48,49,50], further underscoring the limited reliability of Ki-67 for Recurrence Score prediction. Of interest, one study reported that although no significant correlation between Ki-67 and Recurrence Score was observed in the overall study population, a significant association emerged within a predefined subgroup of tumors with high Ki-67 expression (≥30%), suggesting that the relationship between Ki-67 and genomic risk may be restricted to highly proliferative tumors [50]. Beyond issues of statistical correlation, several studies have emphasized that Ki-67 and the Oncotype DX Recurrence Score may assign discordant risk categories at the individual patient level, reflecting fundamental differences in how single marker proliferation and multigene genomic profiling capture tumor biology and inform clinical risk stratification [47,51,52].

The objective of this study is to examine the association between the Ki-67 proliferative index and the Oncotype DX Recurrence Score, and to assess its potential value in predicting the Recurrence Score.

## 2. Materials and Methods

### 2.1. Study Design and Data Collection

This was a retrospective, multicenter cohort study including women diagnosed with early-stage, estrogen receptor (ER)-positive, HER2-negative invasive breast cancer who underwent Oncotype DX^®^ Breast Recurrence Score (RS) testing between 2020 and 2023 across multiple institutions in Greece. Clinical and pathological variables, including age, nodal status, tumor size, histologic grade, Ki-67 proliferation index, and RS results, were collected through comprehensive review of medical records.

### 2.2. Definition of Variables

The Oncotype DX Recurrence Score was analyzed both as a continuous variable and as a categorical variable, using clinically relevant thresholds. Patients were classified into low genomic risk (RS 0–25) and high genomic risk (RS > 25), in accordance with contemporary clinical trial evidence and guideline recommendations.

Ki-67 expression was evaluated using two approaches:

1. A binary classification (<20% vs. ≥20%), based on a frequently recommended and widely used cut-off in clinical practice and research.
2. A three-tier classification defining low (≤5%), intermediate (>5% and <30%), and high (≥30%) Ki-67 expression, as proposed by the International Ki-67 in Breast Cancer Working Group.

Nodal status was categorized as node-negative (N0), micrometastatic nodal involvement (N1mic), or node-positive disease (N1). Histologic grade was assessed according to standard pathology criteria and analyzed as grades 1, 2, or 3.

### 2.3. Statistical Analysis

Descriptive statistics were used to summarize baseline clinical and pathological characteristics. Continuous variables were reported as medians with ranges, and categorical variables as frequencies and percentages. Data processing and visualization were performed using both Python (version 3.12.2) and R (version 4.5.3). In Python, pandas was used for data handling, while matplotlib and seaborn were used to generate heatmaps, scatter plots, and distribution plots. In parallel, additional analyses and visualizations were performed in R using ggplot2 and ggpubr, including violin plots and concordance analyses. Ki-67 was analyzed using predefined categorical groupings (<20% vs. ≥20% and ≤5%, >5–<30%, ≥30%), and RS distributions were assessed with non-parametric tests (Wilcoxon rank-sum and Kruskal–Wallis), with results reported as *p*-values.

### 2.4. Distribution Analyses

The distribution of Recurrence Score categories was assessed across nodal subgroups and Ki-67 expression categories. Comparisons of RS distributions across Ki-67 groups were performed using non-parametric statistical tests, given the non-normal distribution of RS values. These included the Wilcoxon rank sum test for two-group comparisons and the Kruskal–Wallis test for comparisons across more than two groups. Results were visualized using boxplots and distribution plots.

### 2.5. Correlation Analyses

The association between Ki-67 and Recurrence Score was evaluated using Spearman’s correlation coefficient, treating both variables as continuous measures. Correlation analyses were performed in the overall cohort and after stratification by Ki-67 expression categories (binary and three-tier classifications) to assess whether the strength of association differed across proliferative subgroups. Scatter plots were generated to illustrate correlation patterns.

### 2.6. Categorical Association and Concordance Analysis

A categorical association analysis was conducted by cross classifying Ki-67 expression (binary classification: <20% vs. ≥20%) with RS risk categories (0–25 vs. >25). Concordance between Ki-67-defined proliferation categories and RS-based genomic risk classification was calculated as the proportion of patients classified concordantly. Discordant classifications were quantified and reported descriptively. Results were displayed using contingency tables and heatmaps.

### 2.7. Combined Analyses by Histologic Grade and Ki-67

Median Recurrence Scores were evaluated across combinations of histologic grade and Ki-67 expression categories. A heatmap was generated to visualize RS patterns across grade (1–3) and Ki-67 three-tier groups, enabling assessment of the combined impact of tumor differentiation and proliferative activity on genomic risk.

## 3. Results

From 2020 to 2023, a total of 2967 patients in Greece with newly diagnosed, invasive, early-stage breast cancer underwent Oncotype DX Breast Recurrence Score testing across several institutions. Eligible patients had ER-positive, HER2-negative disease and were either node-negative (pN0) or postmenopausal with node-positive disease (pN1), with no evidence of distant metastasis (pM0). Baseline clinical and pathological characteristics of the study population are summarized in Table 1.

In the overall study population (*n* = 2967), the median RS was 16; 2522 patients (85.0%) were categorized in the RS 0–25 group, whereas 445 patients (15.0%) had RS > 25. (Available data in Supplemental File S1).

### 3.1. Comparable Distribution of Recurrence Score Across Nodal Status Groups

Among node-negative patients (N0; *n* = 2636), 2232 patients (84.7%) had an RS of 0–25, while 404 patients (15.3%) had an RS > 25. In patients with micrometastatic nodal disease (N1mic; *n* = 84), 75 patients (89.3%) were classified with RS 0–25 and nine patients (10.7%) with RS > 25. Similarly, among patients with node-positive disease (N1; *n* = 247), 215 patients (87.0%) had RS 0–25 and 32 patients (13.0%) had RS > 25.

**Figure 1:**
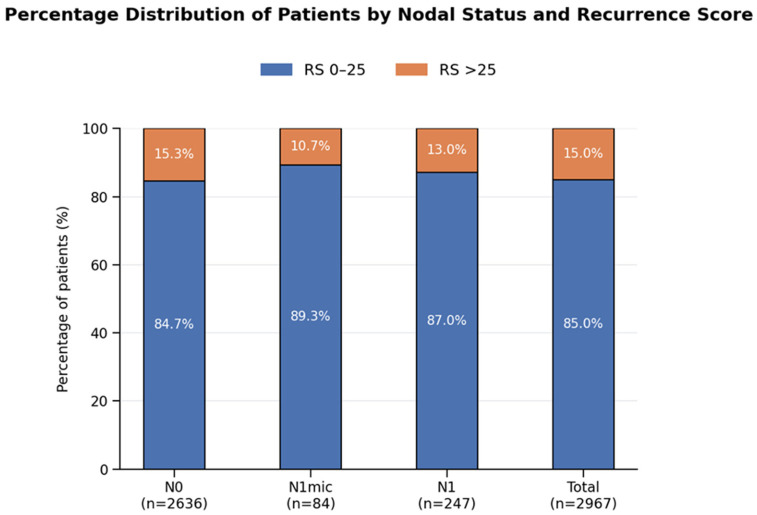
Percentage distribution of patients with low (RS 0–25) and high (RS > 25) genomic risk across nodal subgroups (N0, N1mic, N1) and the total population. Stacked bar charts display the proportion of patients within each Recurrence Score (RS) category for each nodal subgroup (N0, *n* = 2636; N1mic, *n* = 84; N1, *n* = 247) and overall (*n* = 2967). Across all groups, the majority of patients are classified as RS 0–25, with a smaller proportion in the RS > 25 category. The relative distribution of RS categories is broadly similar across nodal subgroups. Sample sizes are indicated on the x-axis. RS = Recurrence Score.

The distribution of Oncotype DX Recurrence Scores was similar across nodal subgroups, with no appreciable differences observed between patients with node-negative, micrometastatic, or node-positive disease (Figure 1).

### 3.2. Modest Correlation Between Ki-67 Expression and Recurrence Score

**Figure 2:**
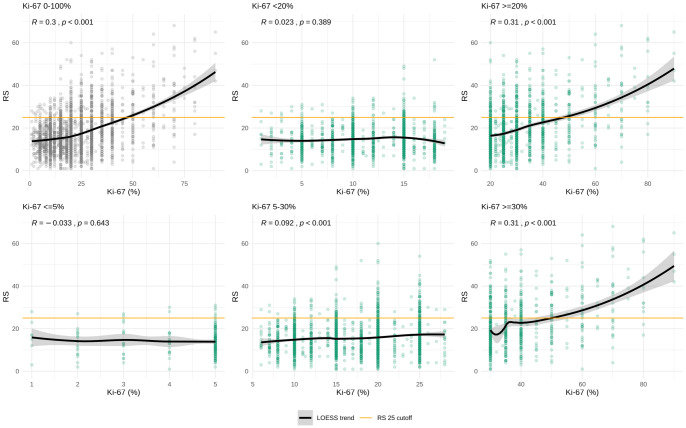
Relationship between Ki-67 expression and Oncotype DX Recurrence Score (RS) in the overall cohort and across Ki-67-defined subgroups. Scatter plots illustrate RS values plotted against Ki-67 (%) for the entire population and within predefined Ki-67 categories (<20%, ≥20%, ≤5%, >5–<30%, and ≥30%). LOESS trend lines (black) and the RS cut-off of 25 (orange line) are shown. Spearman correlation coefficients (R) and *p*-values are indicated in each panel.

Analysis of Ki-67 and Recurrence Score (RS) as continuous variables demonstrated a modest positive correlation in the overall study population (Spearman’s R = 0.3, *p* < 0.001). After stratification by Ki-67 expression using either a binary cut-off (<20% vs. ≥20%) or a three-tier classification (≤5%, >5% to <30%, and ≥30%), a modest positive linear correlation with RS was observed exclusively among tumors with high Ki-67 expression (≥20% or ≥30%, depending on the cut-off applied); whereas, no significant correlation was identified in tumors with low or intermediate Ki-67 expression (Figure 2). The association between Ki-67 and RS remained statistically significant in node-negative disease overall, in postmenopausal node-negative cases, in pre/perimenopausal node-negative cases, and in postmenopausal node-positive subgroups. Specifically, Spearman correlation coefficients were ρ = 0.290 for N0 cases, ρ = 0.268 for postmenopausal N0 cases, ρ = 0.323 for pre/perimenopausal N0 cases, ρ = 0.360 for N1mic cases, and ρ = 0.343 for N1 cases with 1–3 positive nodes (Supplementary Table S1).

### 3.3. Distribution of Recurrence Score Across Ki-67 Categories

**Figure 3:**
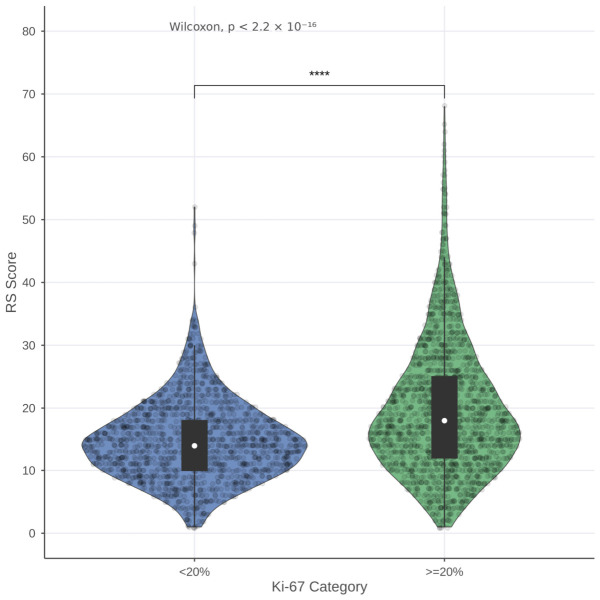
Distribution of Oncotype DX Recurrence Scores (RS) stratified by Ki-67 expression (<20% vs. ≥20%). Violin plots depict the distribution of RS values, with embedded boxplots showing the median and interquartile range. The white dot denotes the median. (Wilcoxon test < 20% vs. ≥20%: **** *p* < 2 × 10^−16^).

**Figure 4:**
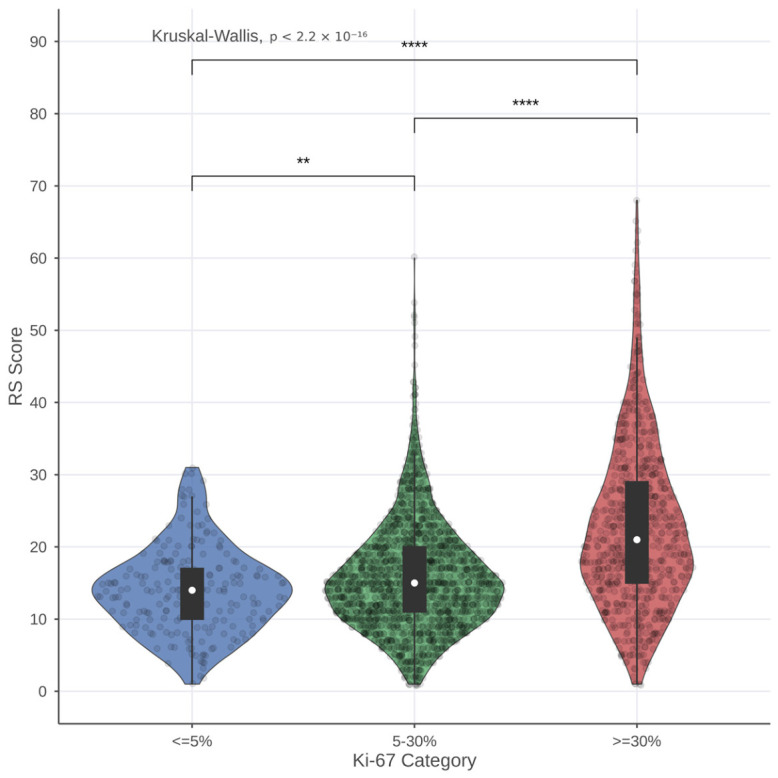
Distribution of Oncotype DX Recurrence Scores (RS) across three-tier Ki-67 expression categories (≤5%, >5–<30%, ≥30%). Violin plots display the distribution of RS values, with embedded boxplots showing the median and interquartile range. The white dot denotes the median. (Global Kruskal–Wallis: *p* < 2 × 10^−16^, Kruskal–Wallis test ≤ 5% vs. 5–30%: ** *p* = 0.0052, Kruskal–Wallis test ≤5% vs. ≥30%: **** *p* < 2 × 10^−16^, Kruskal–Wallis test 5–30% vs. ≥30%: **** *p* < 2 × 10^−16^).

Comparison of Oncotype DX Recurrence Score distributions across Ki-67 expression groups revealed a statistically significant difference in RS distribution, with patients exhibiting higher Ki-67 expression demonstrating a shift toward higher Recurrence Scores. This finding was consistently observed using both the binary (<20% vs. ≥20%) and three-tier (≤5%, >5–<30%, ≥30%) Ki-67 classification systems (Figure 3 and Figure 4).

### 3.4. Marked Discordance Between Ki-67 Categories and Genomic Risk Groups

**Figure 5:**
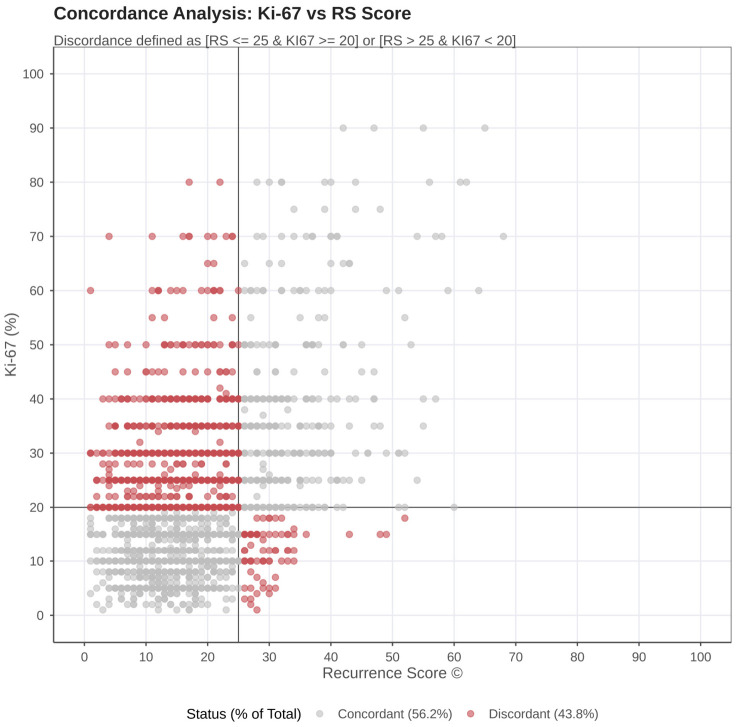
Concordance between Ki-67 expression and Oncotype DX Recurrence Score (RS) at the individual patient level. Each point represents a patient plotted by Ki-67 (%) and RS. Horizontal and vertical lines indicate clinically relevant thresholds (Ki-67 20% and RS 25), dividing patients into concordant and discordant categories. Concordant cases (gray) represent agreement between Ki-67-defined proliferation status and RS-based genomic risk, whereas discordant cases (red) represent mismatched classifications (RS ≤ 25 with Ki-67 ≥ 20% or RS > 25 with Ki-67 < 20%).

In addition to continuous analyses, a categorical association analysis was conducted by stratifying patients according to Ki-67 expression (<20% vs. ≥20%) and Oncotype DX Recurrence Score (RS 0–25 vs. >25) to assess concordance between Ki-67-defined proliferation categories and Recurrence Score-based genomic risk classification. Overall, concordance between Ki-67 status and genomic risk category was observed in 56.2% of cases. Discordant classifications were also identified: 5.6% of patients with low Ki-67 expression (<20%) had a high Recurrence Score (>25); whereas, 76.9% of patients with high Ki-67 expression (≥20%) were classified as low genomic risk (RS 0–25) (Figure 5). Concordance between Ki-67 categorical status and RS risk category was further evaluated across nodal and age-defined subgroups. In node-negative patients, discordance rates were similar in patients ≤50 years and >50 years old, 44.4% and 45.0%, respectively, compared with 44.8% in the overall N0 cohort. In the N1 subgroup, the discordance rate was lower at 36.3%, although this subgroup was smaller and consisted of postmenopausal patients. The predominant discordant pattern was Ki-67 ≥ 20% with RS ≤ 25, indicating that a substantial proportion of tumors classified as highly proliferative by Ki-67 did not correspond to high genomic risk by RS (Supplementary Figure S1).

Based on the three-tier Ki-67 classification, high RS (>25) was observed in 5.4% of patients with low Ki-67 levels, 9.3% of those with intermediate Ki-67 levels, and 33.5% of those with high Ki-67 levels (Table 2).

### 3.5. Higher Histologic Grade and Ki-67 Are Associated with Elevated Recurrence Score

**Figure 6:**
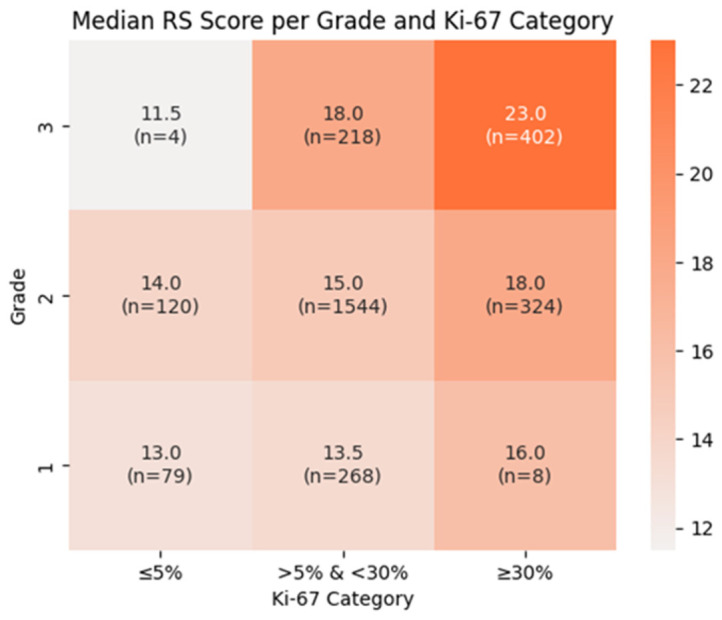
Heatmap illustrating median Oncotype DX Recurrence Score (RS) according to combined histologic grade (grades 1–3) and Ki-67 expression categories (≤5%, >5–<30%, ≥30%). Each cell shows the median RS and the number of patients (*n*).

Analysis of median Oncotype DX Recurrence Score (RS) according to tumor grade and Ki-67 category is presented in Figure 6. Across all histologic grades, median RS values increased with higher Ki-67 expression. A similar trend of increasing RS was observed with increasing tumor grade within each Ki-67 category.

In grade 1 tumors, the median RS increased from 13.0 in the Ki-67 ≤ 5% category to 13.5 in the >5–<30% category and to 16.0 in the Ki-67 ≥ 30% category. In grade 2 tumors, corresponding median RS values were 14.0, 15.0, and 18.0, respectively. The highest RS values were observed in grade 3 tumors, with a median RS of 11.5 in the Ki-67 ≤ 5% group, increasing to 18.0 in the >5–<30% group and to 23.0 in the Ki-67 ≥ 30% group.

Overall, higher Ki-67 expression and higher tumor grade were both associated with higher median Recurrence Scores, with the combination of grade 3 tumors and Ki-67 ≥ 30% demonstrating the highest median RS values.

## 4. Discussion

In this real-world retrospective cohort, we identified a modest overall correlation between Ki-67 and the Oncotype DX Recurrence Score (R = 0.30), in line with prior studies reporting correlation coefficients in the range of 0.3–0.4 [45,53,54]. Importantly, stratification by clinically relevant Ki-67 cut-offs revealed that this association was heterogeneous and largely contingent on higher Ki-67 expression levels. Notably, the 20% threshold has historically been proposed as an optimal cut-off for Ki-67 and continues to be used in clinical trials as an inclusion criterion to identify higher-risk patient populations. However, more recently, the International Ki-67 in Breast Cancer Working Group has recommended a three-tier classification system to improve standardization and clinical interpretation. Specifically, no meaningful correlation was observed among tumors with low proliferative activity (Ki-67 < 20%, Ki-67 ≤ 5%, or Ki-67 > 5% and <30%), whereas a modest association emerged in tumors with elevated Ki-67 expression (Ki-67 ≥ 20% or ≥30%). Similar patterns, in which the association between Ki-67 and genomic risk becomes apparent predominantly at higher proliferative levels, have been reported in other studies [50].

These findings suggest that the observed global correlation is predominantly driven by highly proliferative tumors, while low proliferation tumors exhibit biological heterogeneity that is not adequately captured by Ki-67 alone.

### 4.1. Concordance Analysis: Limited Predictive Performance of Ki-67

The broad dispersion of Recurrence Score (RS) values observed across all Ki-67 strata underscores substantial underlying biological heterogeneity within each Ki-67 category. Using a clinically relevant Ki-67 cut-off of 20%, concordance between Ki-67-defined proliferative status and RS-based genomic risk classification was limited to 56.2%, indicating that Ki-67 accurately reflected genomic risk in just over half of cases. Such a degree of agreement is insufficient to support clinical decision-making and aligns with prior reports highlighting the limited reliability of Ki-67 alone in predicting Recurrence Score categories, with marked discordance across the full range of Ki-67 expression [52]. Notably, 76.9% of tumors with elevated Ki-67 (≥20%) were classified as low genomic risk (RS ≤ 25), while a smaller but clinically meaningful proportion of tumors with low Ki-67 exhibited high Recurrence Scores (5.6%), illustrating the potential for both overtreatment and undertreatment if Ki-67 were used as a surrogate for genomic testing.

Additional insight was provided by analysis using the three-tier Ki-67 classification, which revealed that most tumors (68.4%) clustered within the intermediate Ki-67 range, a category for which prognostic discrimination is limited according to International Ki-67 in Breast Cancer Working Group recommendations [16]. Within the low Ki-67 group (≤5%), only a small fraction of tumors (5.4%) was associated with high Recurrence Scores, whereas in the high Ki-67 group (≥30%), most patients (66.5%) demonstrated low genomic risk (RS 0–25). Collectively, these findings reinforce that Ki-67 and multigene assays capture overlapping but non-equivalent dimensions of tumor biology and should not be regarded as interchangeable tools for individualized risk stratification.

### 4.2. Biological Interpretation: Proliferation Versus Tumor Biology

The observed discordance can be explained by fundamental biological differences between Ki-67 and the Recurrence Score (RS). Ki-67 represents a single-marker assessment of cellular proliferation, whereas RS integrates the expression of 21 genes involved not only in proliferation but also in estrogen signaling, invasion, and other key tumor biology pathways [36]. This distinction is particularly evident in tumors with low Ki-67 expression, where the absence of correlation suggests that non-proliferative biological processes contribute substantially to genomic risk. Conversely, in highly proliferative tumors, Ki-67 contributes more prominently to RS, accounting for the modest correlation observed in this subgroup. Nonetheless, even within highly proliferative tumors, the broad range of RS values indicates that proliferation alone is insufficient to fully define tumor behavior. In addition, well-recognized inter-laboratory variability and lack of analytical standardization further limit the reliability of Ki-67 as a predictive biomarker [16,17,18,19,20,21,22].

### 4.3. High Clinical Risk Does Not Equate to High Genomic Risk

One of the most clinically relevant findings of this study is that patients with traditionally high-risk clinicopathologic features—specifically high Ki-67 (≥30%) and high histologic grade (G3)—frequently exhibited RS values below the chemotherapy treatment threshold. These results reinforce the concept that clinicopathologic and genomic risk are not interchangeable. Similar observations have been reported in real-world cohorts, where tumors with aggressive histopathologic characteristics may nonetheless demonstrate low genomic risk [55,56,57]. Clinically, this suggests that a subset of patients who would historically be considered for chemotherapy based on elevated Ki-67 or tumor grade may safely forgo cytotoxic treatment when therapy is guided by genomic profiling.

### 4.4. Consistency Across Nodal Subgroups

We further observed that RS distributions were comparable across nodal subgroups (N0, N1mic, N1), supporting the notion that RS primarily reflects intrinsic tumor biology rather than anatomic disease burden and suggesting that genomic risk, as assessed by RS, is largely independent of nodal involvement. This finding is consistent with evidence from large prospective trials, including TAILORx and RxPONDER, which demonstrated that the prognostic and predictive value of RS extends across nodal categories, particularly in postmenopausal patients [33,35].

### 4.5. Clinical Implications

Taken together, these findings have important implications for clinical practice. Although Ki-67 is commonly used as a marker of tumor proliferation in clinical practice, its use as a standalone biomarker to guide adjuvant therapy decisions is limited by significant inter-observer variability, lack of assay standardization, and inconsistent cut-off definitions. In contrast, the Recurrence Score (RS) and other multigene genomic assays provide a more robust and reproducible assessment by integrating the expression of multiple genes involved in key biological pathways, including proliferation, hormone receptor signaling, and invasion. Importantly, Oncotype DX has been prospectively validated in large randomized clinical trials, demonstrating their ability not only to stratify recurrence risk but also to predict the likelihood of benefit from adjuvant chemotherapy in specific early-stage breast cancer populations. By comparison, Ki-67 has not been validated in randomized trials as a predictive biomarker for chemotherapy benefit. This comprehensive molecular profiling therefore enables more accurate risk stratification and supports more informed, individualized treatment decisions. While Ki-67 may provide complementary information, RS can refine risk assessment beyond proliferation alone and offer clinically actionable guidance for therapy selection.

Our results support the continued use of validated multigene assays in patients with HR-positive, HER2-negative early breast cancer, particularly in scenarios where clinicopathologic features suggest high risk but treatment decisions remain uncertain. This consideration is especially relevant in healthcare settings where access to genomic testing may be limited due to factors such as cost, lack of reimbursement, limited laboratory infrastructure, or delayed turnaround times. In these contexts, clinicians may rely more heavily on readily available surrogate biomarkers, such as Ki-67, alongside traditional clinicopathologic features to inform treatment decisions. However, while such approaches may offer practical value, they inherently lack the biological depth, standardization, and predictive validation of multigene genomic assays. As a result, reliance on surrogate markers alone may lead to less precise risk stratification and potential over- or under-treatment. These challenges highlight the need to interpret surrogate biomarkers with caution and underscore the importance of improving access to validated genomic tools to ensure more consistent and evidence-based decision-making across diverse healthcare environments.

### 4.6. Limitations

This study has several limitations First, Ki-67 assessment was not centrally reviewed, introducing potential inter-laboratory variability, a well-recognized limitation of Ki-67 interpretation. While this heterogeneity may contribute to variability in the results, it reflects routine clinical practice across institutions and may therefore enhance the external validity and generalizability of our findings. Second, the retrospective design carries inherent risk of selection bias. Third, certain biologically discordant subgroups (e.g., G1 tumors with high Ki-67 or G3 tumors with low Ki-67) were underrepresented, reflecting real-world distributions but limiting the robustness of subgroup analyses.

## 5. Conclusions

In this large real-world cohort, Ki-67 demonstrated a modest correlation with RS, driven predominantly by tumors with high proliferative activity. However, the substantial discordance observed across clinically relevant Ki-67 thresholds indicates that Ki-67 cannot serve as a surrogate for genomic testing. These findings reinforce the critical role of multigene assays in accurately stratifying recurrence risk and guiding adjuvant treatment decisions in early-stage HR-positive/HER2-negative breast cancer.

## Figures and Tables

**Table 1:** Clinical characteristics of the study population.

Characteristic	
Median age (range)-yr	56 (21–85)
Age—no. (%)	
≤50	990 (33.4)
>50	1977 (66.6)
Nodal Status—no. (%)	
N0	2636 (88.9)
N1mic	84 (2.8)
N1- 1 positive lymph node	171 (5.8)
N1- 2 positive lymph nodes	60 (2.0)
N1- 3 positive lymph nodes	16 (0.5)
Median tumor size (range)-cm	1.5 (0.2–10.2)
Tumor size—no. (%)	
T1a (≤0.5 cm)	61 (2.1)
T1b (>0.5 cm and ≤1 cm)	646 (21.8)
T1c (>1 cm and ≤2 cm)	1551 (52.2)
T2 (>2 cm and ≤5 cm	678 (22.9)
T3 (>5 cm)	31 (1)
Histologic grade at diagnosis—no. (%)	
1	355 (12.0)
2	1988 (67.0)
3	624 (21.0)
Median Ki-67 (%)	20
Ki-67—no. (%)	
Binary classification	
<20%	1378 (46.4)
≥20%	1589 (53.6)
Three-tier classification	
≤5%	203 (6.8)
>5% and <30%	2030 (68.4)
≥30%	734 (24.8)

**Table 2:** Distribution of Oncotype DX Recurrence Score categories across three-tier Ki-67 expression groups.

	*N* (%)
Ki-67 ≤ 5%	203
-RS 0–25	192 (94.6)
-RS > 25	11 (5.4)
Ki-67 >5% and <30%	2030
-RS 0–25	1842 (90.7)
-RS > 25	188 (9.3)
Ki-67 ≥ 30%	734
-RS 0–25	488 (66.5)
-RS > 25	246 (33.5)

## Data Availability

Data derived from patients’ anonymized medical records are available in Supplemental File S1.

## Supplementary Materials

The following supporting information can be downloaded at: https://www.mdpi.com/article/10.3390/cancers18111731/s1, Figure S1: Concordance between Ki-67 and Recurrence Score according to nodal and age subgroups. Scatter plots showing Ki-67 percentage versus 21-gene Recurrence Score in the overall node-negative cohort, node-negative patients ≤ 50 years, node-negative patients > 50 years, and patients with N1 disease. Vertical and horizontal reference lines indicate RS = 25 and Ki-67 = 20%, respectively. Discordance was defined as RS ≤ 25 with Ki-67 ≥ 20%, or RS > 25 with Ki-67 < 20%. Red points indicate discordant cases, and grey points indicate concordant cases. Discordance rates were similar across node-negative age subgroups, while the N1 subgroup showed a lower discordance rate, although this analysis should be interpreted cautiously due to smaller sample size; Table S1: Distribution of Recurrence Score and Ki-67 and Their Spearman Correlation Across Nodal and Menopausal Subgroups; File S1: Evaluating the Association of Ki-67 with Oncotype DX.

## Abbreviations

The following abbreviations are used in this manuscript:

**Abbreviation**	**Full Name**
Ki-67	Ki-67 proliferation index (Ki-67 nuclear antigen)
ER	Estrogen receptor
PR	Progesterone receptor
HER2	Human epidermal growth factor receptor 2
HR	Hormone receptor
RS	Recurrence Score
Oncotype DX	Oncotype DX Breast Recurrence Score assay
MKI-67	Marker of proliferation Ki-67 gene
pM0	Pathologic absence of distant metastasis
pN0	Pathologic node-negative disease
pN1	Pathologic node-positive disease
N0	Node-negative disease
N1	Node-positive disease
N1mic	Micrometastatic nodal involvement
G1	Histologic grade 1
G2	Histologic grade 2
G3	Histologic grade 3
pCR	Pathological complete response
IKWG	International Ki-67 in Breast Cancer Working Group
ESMO	European Society for Medical Oncology
ASCO	American Society of Clinical Oncology
NCCN	National Comprehensive Cancer Network
IBCSG	International Breast Cancer Study Group
TAILORx	Trial Assigning Individualized Options for Treatment (Rx)
RxPONDER	Rx for Positive Node, Endocrine Responsive Breast Cancer
monarchE	monarchE clinical trial
R	Spearman correlation coefficient
*p*	*p*-value
