# Supplementary material for: Evaluating the Association of Ki-67 with Oncotype DX Recurrence Score in Early-Stage ER-Positive/HER2-Negative Breast Cancer
Source: Cancers (Basel). 2026 May 26;18(11):1731. doi: 10.3390/cancers18111731 (PMC13255838; doi:10.3390/cancers18111731)
Supplement: Supplementary file 1 [file cancers-18-01731-s001.zip › Supplementary Figure S1.pdf]

Supplementary Figure S1

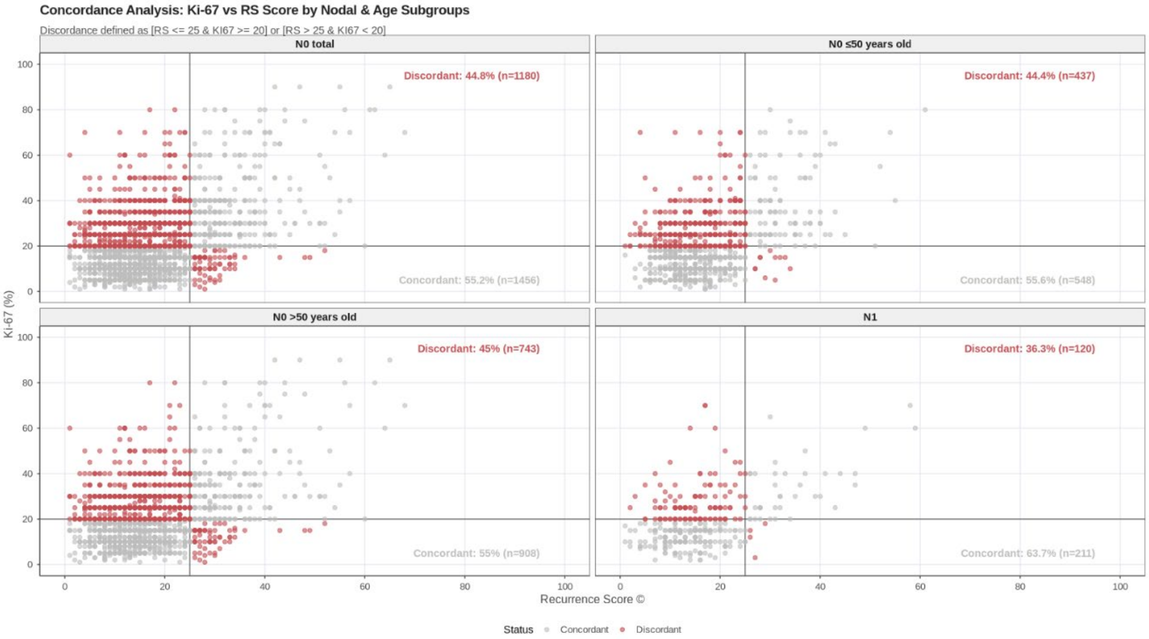

Supplementary Figure S1. **Concordance between Ki-67 and Recurrence Score according to nodal and age subgroups.** Scatter plots showing Ki-67 percentage versus 21-gene Recurrence Score in the overall node-negative cohort, node-negative patients ≤ 50 years, node-negative patients > 50 years, and patients with N1 disease. Vertical and horizontal reference lines indicate RS = 25 and Ki-67 = 20%, respectively. Discordance was defined as RS ≤ 25 with Ki-67 ≥ 20%, or RS > 25 with Ki-67 < 20%. Red points indicate discordant cases, and grey points indicate concordant cases. Discordance rates were similar across node-negative age subgroups, while the N1 subgroup showed a lower discordance rate, although this analysis should be interpreted cautiously due to smaller sample size.
